# Real-Life Data of Patients with Exudative Age-Related Macular Degeneration

**DOI:** 10.14744/bej.2021.63496

**Published:** 2021-12-17

**Authors:** Mesut Togac, Ekrem Celik, Evrim Polat, Sibel Ahmet, Ahmet Alperen Koc, Zeynep Alkin

**Affiliations:** 1. Department of Ophthalmology, Tekirdag City Hospital, Tekirdag, Turkey; 2. Department of Ophthalmology, Catalca Ilyas Cokay State Hospital, Istanbul, Turkey; 3. Department of Ophthalmology, Yeni Yuzyil University Faculty of Medicine, Istanbul, Turkey; 4. Department of Ophthalmology, Dunyagoz Hospital, Istanbul, Turkey

**Keywords:** Exudative age-related macular degeneration, intravitreal injection, real-life data

## Abstract

**Objectives:**

The aim of this study was to examine and provide real-life data of patients with exudative-type age-related macular degeneration (AMD).

**Methods:**

A total of 189 eyes of 160 patients with exudative AMD treated with intravitreal anti–vascular endothelial growth factor therapy (anti-VEGF) injections (ranibizumab 0.3 mg/0.05 mL, aflibercept 2 mg/0.05 mL, bevacizumab 1.25 mg/0.05 mL) were included in the study. Patient demographic characteristics, and details of the clinical examinations, number of injections, best-corrected visual acuity (BCVA) measured with the Snellen chart, optical coherence tomography, and fundus fluorescein angiography images were evaluated at the first visit and during the follow-up period.

**Results:**

In the study group, 78 of the patients were female (48.8%) and 82 were male. The mean age was 72.20±8.97 years. While no treatment had been applied to 151 eyes before the first examination, 38 had previously received an intravitreal injection at another center. The mean number of patient visits was 5.83 in the first year, 4.68 in the second year, and 3.84 in the third year, and the mean number of injections was 4.70 in the first year, 2.08 in the second year, and 1.51 in the third year. The mean BCVA change between the first visit and the first, second, and third years was not statistically significant (p>0.05), and a significant change was observed in the mean central macular thickness (p<0.05).

**Conclusion:**

Anatomical and functional success was achieved with anti-VEGF treatment after fewer injections and visits than have been reported in randomized, controlled, clinical studies in the literature. The number of injections and visits recorded in this study was consistent with the data of other real-life studies.

## Introduction

Age-related macular degeneration (AMD) is the most common cause of central vision loss in people over 65 years of age in developed countries (1, 2). Over the age of 65, the prevalence ranged from 1.2 to 3.8%, while over 75 years old, it was between 19.7% and 36.8% (3). The disease primarily affects the choriocapillaris, bruch membrane, and retinal pigment epithelium (RPE), and there are 2 types of AMD: dry (non-neovascular) and wet (neovascular). Dry type includes abnormalities of the RPE such as drusen, geographic atrophy, areas of non-geographic atrophy, and focal areas of hyperpigmentation in the macula. The exudative form, which includes most of the cases with severe vision loss, is characterized by choroidal neovascularization (CNV), serous or hemorrhagic detachment of the RPE, and disciform scar (3, 4). Neovascular proliferation extending from the choriocapillaries to the Bruch’s Membrane and filling the space under the RPE causes serous fluid leakage and bleeding (5). Wet type is seen only in 10–20% of all AMD cases, but is responsible for 80–90% of irreversible central vision loss (6, 7).

It has been reported that vascular endothelial growth factor (VEGF) plays a key role in the pathogenesis of CNV with an increase in vascular permeability with neovascularization and fluid accumulation in the retina and under it. VEGF inhibition has been emphasized in the treatment and agents that block VEGF activity have been developed by the researchers. bevacizumab (Avastin/Altuzan, Genentech), ranibizumab (Lucentis, Genentech), and aflibercept (Eylea, Bayer) preparations have widespread clinical applications as intravitreal injection. Numerous controlled, prospective, studies with large case numbers examining CNV treatment due to AMD have reported the treatment with intravitreal anti VEGF agents as a highly effective and safe treatment (8-11). However, since there are no large, controlled, prospective studies examining the treatment with anti VEGF agents in the treatment of CNV due to other reasons, case reports and case series in the literature provide guidance in the treatment of these cases (12).

Our aim in this study to reveal the clinical follow-up and real life data of patients who diagnosed by exudative AMD and received intravitreal injection therapy in a tertiary retinal center.

## Methods

We analyzed records of 189 eyes of 160 patients who were diagnosed with exudative AMD and received intravitreal anti-VEGF (ranibizumab/aflibercept/bevacuzimab) injection therapy at the retina center of Istanbul Beyoglu Eye Training and Research Hospital retrospectively. The study procedure adhered to the Declaration of Helsinki and approved by the Institutional Ethics Committee (E-17091262-929). Cornea problems, cataracts, lens problems, glaucoma, eyes with CNV developing due to retinal diseases other than exudative type AMD, or other retinal diseases such as pathological myopia, angioid streaks or ocular histoplasmosis, were excluded from the study. Patients with uncontrolled hypertension, diabetes mellitus, impaired bleeding profile, renal dysfunction, and a history of thromboembolic disease were excluded from the study because they may affect retinal pathology.

Diagnostic fundus fluorescein angiographies of each patient were taken before the treatment. Pre-treatment and follow-up optical coherence tomography (OCT) changes, Snellen and logarithm of minimum angle of resolution (logMAR) charts, best-corrected visual acuity (BCVA) changes and demographic characteristics were evaluated. Anterior segment and examination were performed with a slit lamp, intraocular pressures were measured with Goldman applanation tonometer, and dilated fundus examinations were performed with a 90 D lens. Anti-VEGF treatment was initiated after the diagnosis of exudative AMD was made, and anti-VGEF treatment was applied as needed for each patient according to the evaluations of examination primarily by adopting a pro re nata (PRN) regimen. Activity and related re-injection criteria in CNV were determined as a 1-line decrease in visual acuity on the ETDRS chart, detection of an increase of 100 μm or more in central foveal thickness compared to the last examination, the formation of subretinal hemorrhage foci that did not exist before, development or increase of intra-subretinal fluid. Before intravitreal anti VEGF injection, surgical hand disinfection was performed, sterile gloves were applied, and regional area cleaning was performed with 10% povidone iodine. Then, topical anesthesia was provided with proparacaine hydrochloride, and 5% povidone iodine drops were applied to the eye for endophthalmitis prophylaxis. The eyelids were covered with a sterile dressing, and a speculum was inserted to open the eyelid. A 27 gauge injector needle inserted 3.5 mm away from the limbus temporally in the pseudophakic eye and 4 mm in the phakic eye. Eye closure tapes of the patients were removed 2 h after the application and topical antibiotic treatment was started with one drop every hour. Controls were made on the 1^st^ day after injection and the antibiotic dose was adjusted according to the patient’s examination findings. All patients were called for routine control at the 1^st^ week, 1^st^ month, 3^rd^ month, and were warned to come immediately in case of burring, decreased vision and pain.

All statistical analyzes were performed with SPSS Statistics 20.0 for Windows software (SPSS, Inc. IBM Corp., Armonk, NY, USA). Mean, standard deviation, median, minimum, maximum, ratio, and frequency values were used in the descriptive statistics of the data. The distribution of variables was checked with the Kolmogorov–Smirnov Test. In the statistical analysis of the comparison of quantitative data, the dependent sample t-test was used for variables with normal distribution, and the Wilcoxon test was used for variables that did not show normal distribution.

## Results

The mean age of the patients was 72.20±8.97 and 78 (48.8%) of the patients were female and 82 (51.3%) were male. While the lesion was detected on the right side in 93 patients (49.2%), it was detected on the left side in 96 patients (50.8%). While 151 (79.9%) of the patients included in the study had not received intravitreal injection treatment before, 38 (20.1%) patients had received intravitreal injection treatment in the centers they applied previously. The treatment protocol applied to the patients was 3 + PRN in 170 (89.9%) patients and 1 + PRN in 19 (10.1%) patients. When the number of visits of per year were evaluated, at the first year was 5.83±1.73, in the second year was 4.68±2.19, and at the third year was 3.84±2.43.

**Figure 1.**
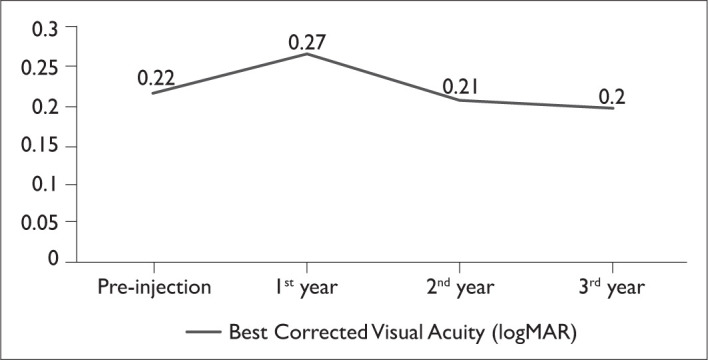
The mean visual acuity changes in pre-injection, first, second and third year visits.

Considering the number of injections per year, it was 4.70±2.39 at the 1^st^ year, 2.08±1.89 at the 2^nd^ year, and 1.51±2.43 at the 3^rd^ year. While the BCVA (logMAR) of our patients was 0.99±0.68 at the pre-injection, it was evaluated as 0.94±0.68 after the loading dose, and this change was not statistically significant (p=0.165). When the yearly changes in visual acuity are evaluated, the mean BCVA at pre-injection is 0.22±0.22, at 12 months 0.27±0.42, at 24 months 0.21±0.21, at 36 months 0.20 + 0.25, and this change was not statistically significant (p>0.05) (Fig. 1).

**Figure 2.**
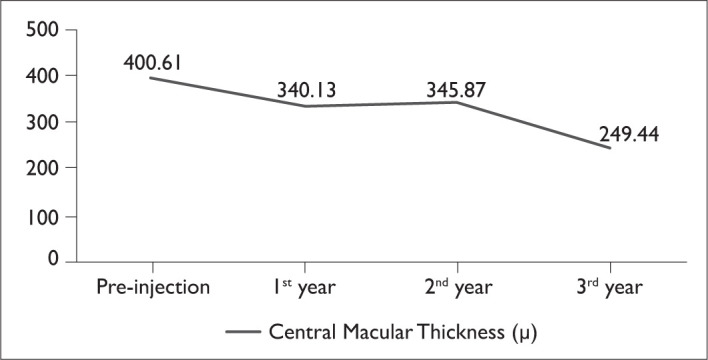
The mean central macular thickness changes in pre-injection, first, second and third year visits.

When the change in mean central macular thickness was examined, it was 400.61±141.31 at pre-injection, 340.13±113.21 at 12 months, 345.87±132.60 at 24 months and 249.44±142.58 at 36 months, and this was statistically significant (p<0.05) (Fig. 2).

## Discussion

The incidence and prevalence of ecudative AMD shows a significant increase with age, and the follow-up of patients and compliance with the frequency of injections can be difficult. It is very important to determine an appropriate treatment plan in harmony with the patients and to ensure that this plan is maintained without interrupting follow-up and intravitreal injections (13-15).

Different results have been reported in studies conducted to determine the relationship of YBMD with gender; While there was no significant gender difference in AMD prevalence in the Blue Mountains Eye Study study groups, studies conducted in Japan reported that early data had higher prevalence and incidence of AMD in men (16, 17). On the contrary, it was reported that prevalence and incidence were higher in women in The Third National Health and Nutrition Examination Survey and Beaver Dam Eye Study groups (18, 19). In our study, there was no significant difference between the genders in terms of prevalence and incidence of male patients (51.3%) and female patients (48.8%).

In the ANCHOR study on ranibizumab for exudative AMD treatment, the patients with predominant classical subfoveal lesion secondary to AMD were selected and 143 patients who received photodynamic therapy (PDT), 140 patients who received 0.5 mg/ml ranibizumab and 140 patients who were received 0.3 mg/ml were compared and visual acuity was compared in terms of stabilization percentages at the end of the 1^st^ and 2^nd^ year. In the PDT group, patients received PDT every three months if necessary and sham injection once a month. The patients in the ranibizumab group received intravitreal ranibizumab every month and sham PDT every three months. Visual acuity increased by more than 15 letters in 40% of eyes treated with 0.5 mg/ml ranibizumab in the 12^th^ month. In this group, the proportion of eyes with increased visual acuity and a decrease of <15 letters was found to be 96%. In eyes treated with 0.5 mg/ml ranibizumab, mean visual acuity increased by 11.3 letters. In the second year results of the same study, it was reported that ranibizumab preserved the visual level in 90% of the dominant classical lesions (20). In the MARINA study, the efficacy of ranibizumab in minimal classical/occult lesions was investigated; For this purpose, 238 patients received ranibizumab 0.5 mg/ml, 238 patients 0.3 mg/ml ranibizumab and 240 patients received sham injection. Intravitreal injections were received every month, and the results of the patients were evaluated at the end of the 1^st^ and 2^nd^ year. At the end of the first year, it was found that the visual level was preserved in 95% of the eyes treated with 0.5 mg/ml ranibizumab, and there was at least 15 letters increase in the visual level in 34%. In the control group, it was observed that the vision level was preserved in 62% of the eyes and at least 15 letters increase in 5%. In the 0.5 mg/ml ranibizumab group, mean visual acuity increased by 7.2 letters, while in the control group, there was an average decrease of 10.4 letters. It was observed that the effect of ranibizumab on maintaining visual acuity continued in the second year of the study. At the end of the second year, it was determined that 90% of the eyes treated with 0.5 mg/ml ranibizumab preserved the visual level and 33% had an increase of at least 15 letters in the visual level. In the control group, it was observed that the vision level was preserved in 53% of the eyes and at least 15 letters increase in 4%. In the 0.5 mg/ml ranibizumab group, mean visual acuity increased by 6.6 letters, while the mean visual acuity decreased by 14.9 letters in the control group (21). In the PIER study, unlike the other two studies, all lesion types were included in the study and a treatment program was made in which the number of injections was reduced. For this purpose, 184 eyes with occult/minimal classical/dominant classical lesions were included in the study and 60 patients received 0.3 mg/ml ranibizumab, 61 patients received 0.5 mg/ml ranibizumab, and 63 patients received sham injection monthly for the first 3 months and then every 3 months been applied. At the end of the 1^st^ year, 13% of the patients who received 0.5 mg/ml ranibizumab had an increase of more than 15 letters and 90% preserved their vision, while the rate of patients with preserved vision in the control group was found to be 49%. Mean visual acuity decreased by 0.2 letters in the ranibizumab group, while a decrease of 16.3 letters was found in the sham group (22). In the PrONTO study, the effect of reducing the number of injections on drug efficacy was investigated. Therefore, ranibizumab 0.5 mg/ml was administered every month for the first 3 months in 40 eyes with occult/minimal classical/dominant classical lesions, and then the injection was repeated according to visual acuity, OCT and FFA findings. At the end of the 1^st^ year, it was determined that the vision level was preserved in 95% of the eyes, and there was at least 15 letters increase in 35%. At the end of the 2^nd^ year, it was observed that the vision level was preserved in 97% of the eyes, and there was at least 15 increase in 43%. At the end of the 2^nd^ year, an average of 9.9 injections were given and visual acuity increased by an average of 11.1 letters (10). The mean number of injections in the 1^st^ year of the ANCHOR, MARINA, PIER, and PrONTO studies were reported as 13, 13, 6, and 5.5, respectively. In ANCHOR and MARINA studies, it has been shown that intravitreal ranibizumab is effective in all types of neovascular AMD when administered monthly. In these two pilot studies, it was shown that visual acuity was best achieved with monthly continuous injections following the first three doses. However, it is not possible to continue monthly injections in real life, instead, follow-up of patients according to lesion activity and flexible treatment protocols that can be applied when necessary are recommended (10, 20-22).

The LUMINOUS study is a multicenter retrospective study conducted to evaluate the real-world safety and efficacy of ranibizumab in routine clinical practice in the treatment of exudative AMD. In the LUMINOUS study, in which real world data were evaluated, the average number of injections was found to be between 4.3 and 5.5 (8). Similarly, in the AURA study, which revealed current life data on the safety and efficacy of ranibizumab in clinical practice in the treatment of exudative AMD, the mean number of clinical visits and injections at 2 years follow-up was 18.4 and 9.0, respectively (23). In the study conducted by Holz et al. (9) to evaluate the real-life data of anti-VEGF treatment in many countries in exudative AMD, the number of visits of the patients was found as an average of 8.6 in the 1^st^ year and 6.4 in the 2^nd^ year, and the number of injections was found average of 5.0 in the 1^st^ year and 2.2 in the 2^nd^ year. In the study conducted by Beykin et al. (24) in which real-life data of the long-term results of bevacizumab treatment in exudative AMD were evaluated, the number of injections was determined as 5.4 injections. As a matter of fact, when we compare the number of injections with these studies, our number of injections was 4.7 in the 1^st^ year and 2.1 in the 2^nd^ year, and it was similar to these studies. Our number of visits was 5.8 in the 1^st^ year and 4.68 in the 2^nd^ year, which was slightly lower than similar studies.

Exudative AMD is a chronic, progressive disease causing vision loss in the elderly population and regular follow-up is essential. Although anatomical and functional success was achieved with intravitreal anti-VEGF treatment in our study, the increase in visual acuity, the average number of injections and the number of visits were found to be lower compared to randomized, controlled, clinical studies in the literature. However, when compared with the data of real life studies, our injection and visit numbers were found to be compatible with these studies. Considering the clinical conditions and life styles of the patients, our results reflect more real-life data rather than controlled pilot studies and new real-life studies are needed to find optimal follow-up and treatment regimens applicable in current clinical conditions.

### Disclosures

**Ethics Committee Approval:** Istanbul Beyoglu Eye Training and Research Hospital Ethics Commitee E-17091262-929.

**Peer-review:** Externally peer-reviewed.

**Conflict of Interest:** None declared.

**Authorship Contributions:** Involved in design and conduct of the study (MT, EC, EP, SA, AAK, ZA); preparation and review of the study (MT, EC, ZA); data collection (MT, EC, SA, AAK, ZA); and statistical analysis (MT, EP, SA, AAK, ZA).
